# HDAC10 promotes angiogenesis in endothelial cells through the PTPN22/ERK axis

**DOI:** 10.18632/oncotarget.18130

**Published:** 2017-05-24

**Authors:** Baoyu Duan, Dan Ye, Songcheng Zhu, Wenwen Jia, Chenqi Lu, Guiying Wang, Xudong Guo, Yangyang Yu, Chuanyue Wu, Jiuhong Kang

**Affiliations:** ^1^ Clinical and Translational Research Center of Shanghai First Maternity and Infant Health Hospital, Shanghai Key Laboratory of Signaling and Disease Research, Collaborative Innovation Center for Brain Science, School of Life Science and Technology, Tongji University, Shanghai 200092, China; ^2^ Department of Biostatistics and Computational Biology, State Key Laboratory of Genetic Engineering, School of Life Sciences, Fudan University, Shanghai 200433, China; ^3^ Department of Biology and Shenzhen Key Laboratory of Cell Microenvironment, Southern University of Science and Technology, Shenzhen 518055, China; ^4^ Department of Pathology, School of Medicine, University of Pittsburgh, Pittsburgh, PA 15261, USA

**Keywords:** angiogenesis, histone deacetylase 10 (HDAC10), protein tyrosine phosphatase non-receptor type 22 (PTPN22), ERK1/2 phosphorylation, tube formation

## Abstract

Angiogenesis is crucially involved in many physiological and pathological processes including tumor growth, but the molecular mechanisms regulating angiogenesis are incompletely understood. In this study, we investigated the functions and mechanism of histone deacetylase 10 (HDAC10), a member of the HDAC II family, in regulation of angiogenesis. HDAC10 overexpression in human umbilical vein endothelial cells (HUVECs) promoted tube formation, whereas depletion of HDAC10 from HUVECs inhibited tube formation *in vitro* and *in vivo*. Mechanistically, HDAC10 overexpression increased extracellular-regulated kinase 1/2 (ERK1/2) activation, whereas depletion of HDAC10 inhibited ERK1/2 activation. Finally, HDAC10 promoted ERK1/2 phosphorylation by deacetylating the promoter of protein tyrosine phosphatase, non-receptor type 22 (PTPN22) and inhibiting the expression of PTPN22, which is a negative regulator of ERK phosphorylation. Collectively, our results identify HDAC10 as a key regulator of angiogenesis and reveal that HDAC10 functions in this process by binding and deacetylating the PTPN22 promoter and subsequently inhibiting PTPN22 expression, which in turn increases ERK1/2 phosphorylation. Our studies suggest that HDAC10 is a potential target for therapeutic intervention to inhibit angiogenesis and tumor growth.

## INTRODUCTION

Angiogenesis is a critical and complex biological event in many physiological and pathological processes, such as tumor growth [1–3]. This progress relies on the coordinated expression of many genes encoding proteins and angiogenic growth factors that activate endothelial cells and lead to extracellular matrix remodeling, endothelial cell migration and proliferation, capillary tube formation, and subsequent maturation of the new blood vessel [4–7]. Identifying the proteins that regulate angiogenesis and determining the signaling pathways through which they function are therefore of broad physiological and pathological significance.

Studies of the signaling pathways in angiogenesis have revealed several potential agents that regulate angiogenesis [8]. Celastrol, which is derived from a traditional Chinese medicinal plant, inhibits vascular endothelial growth factor (VEGF)-induced VEGF receptor (VEGFR) 2 signaling through the AKT/mammalian target of rapamycin (mTOR)/P70S6K signaling pathway and suppresses angiogenesis [9]. Usnic acid (UA), an active compound that is mainly found in lichens, hinders VEGFR2-mediated extracellular-regulated kinase 1/2 (ERK1/2) and AKT/P70S6K signaling pathways in endothelial cells [10]. In addition, small molecular inhibitors of VEGF2, such as YLL545, may inhibit ERK1/2 phosphorylation and block angiogenesis [11]. A previous study showed that ERK1/2 phosphorylation promotes angiogenesis through Ras-RAF-Erk signaling [12]. Eliceiri et al. have also reported that sustained ERK activity is critical for angiogenesis via integrin *α*v*β*3 [13]. Additionally, adrenomedullin promotes angiogenesis in HUVECs, which can be blocked with an ERK1/2 inhibitor [14]. Altogether, these studies indicate that ERK1/2 plays a pivotal role in angiogenesis.

Protein tyrosine phosphatases (PTPases) remove the phosphate group from phosphorylated tyrosine residues on proteins such as SRCs and ERKs [15]. Based on their cellular localization, PTPases can be divided into two classes, receptor-like and non-receptor. Protein tyrosine phosphatase non-receptor type 22 (PTPN22), which is a member of the protein tyrosine phosphatase non-receptor type family [16], is highly enriched in lymphoid tissues, which regulates ERK1/2 activation. Loss of PTPN22 can enhance mitogen-activated protein kinase (MAPK) activity and promote ERK phosphorylation [17–19]. A previous study showed that PTPN22 is significantly mutated in breast cancer [20]. Additionally, Napolioni et al. reported that the PTPN22 1858C>T (R620W) polymorphism plays a pivotal role in human longevity [21]. However, the proteins that regulate PTPN22 expression and the role of PTPN22 in angiogenesis remain unclear.

Histone acetylation is highly involved in transcriptional regulation and is catalyzed by two kinds of enzymes: histone acetyltransferases (HATs) and histone deacetyltransferases (HDACs) [22]. In general, HDACs repress and silence transcription, and they consist of three different classes (I, II, III). Class I HDACs include HDAC1, 2, 3 and 8; class II HDACs include HDAC4, 5, 7 6, 9, and 10; whereas the class III family include sirtuins1-7 [23]. Previous studies have suggested that certain HDACs regulate endothelial cell behavior and angiogenesis [5, 24–26]. For example, forced expression of HDAC1 promotes angiogenesis of HUVECs by downregulating p53 and von Hippel–Lindau tumor suppressor [27]. Hulsurkar et al. reported that HDAC2 is indispensable for β-adrenergic signaling-induced angiogenesis [28]. In contrast, HDAC5 can bind to the promoter regions of the FGF2 and Slit2 genes, inhibiting their expression and disrupting endothelial cell angiogenesis [29]. HDAC10 was isolated and characterized as a class II HDAC, and it exerts its biological functions in several processes, including autophagy, cell cycle, and DNA mismatch repair [30–34]. Our previous studies suggest that HDAC10 suppresses cervical cancer metastasis by inhibiting matrix metalloproteinase 2 and 9 expression [35]. The function of HDAC10 in angiogenesis, however, is unknown. In the current study, we provide evidence showing that HDAC10 is an important positive regulator of angiogenesis and that it functions in this process through regulating the PTPN22-ERK1/2 signaling axis.

## RESULTS

### HDAC10 promotes angiogenesis *in vitro* and *in vivo*

**Figure 1:**
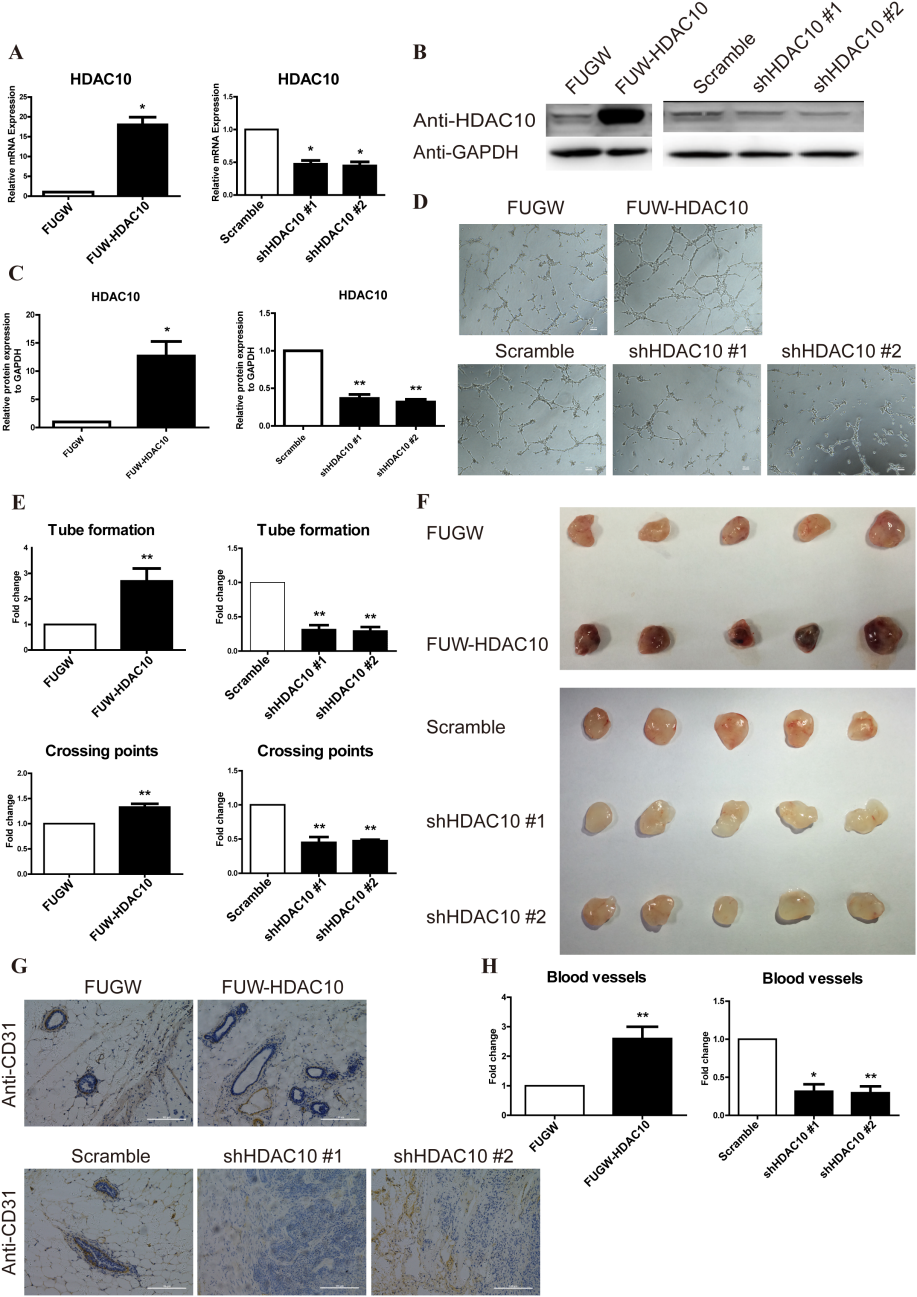
HDAC10 is required for angiogenesis *in vitro* and *in vivo* HUVECs were infected with FUW-HDAC10, the control FUGW, shHDAC10#1, shHDAC10#2 or the scrambled RNA control lentiviruses. **(A)** HDAC10 mRNA expression was analyzed by real-time PCR. **(B)** HDAC10 protein expression was analyzed by Western blotting. **(C)** Quantitative analysis of the Western blots in **(B)**. **(D)** The *in vitro* formation of HUVEC tubes on Matrigel was performed as described in the “Materials and Methods”. **(E)** Quantitative analysis of the tubes and crossing points in panel D. **(F)** The effect of HDAC10 on *in vivo* angiogenesis assessed by a Matrigel plug assay (n = 5 per group). Photographs of representative Matrigel plugs show vessel growth and CD31 expression **(G)**. **(H)** The blood vessels were quantified by manually counting the images shown in **(G)**, and the results are presented as the percentage of the control (n = 5). Data are presented as the means ± standard errors of the means (SEM). *P < 0.05 and **P < 0.01 compared with the control or scrambled control.

We designed lentivirus vector plasmids containing the HDAC10 cDNA and two different shRNAs targeting the HDAC10 sequence to evaluate the role of HDAC10 in angiogenesis *in vitro*. Lentiviral particles containing the HDAC10 cDNA or the shRNAs were used to infect HUVECs. Overexpression and knockdown of HDAC10 in the corresponding viral infectants were confirmed by real-time PCR (Figure 1A) and Western blotting (Figure 1B-1C).

The tube formation assay was utilized to evaluate the role of HDAC10 in regulation of angiogenesis *in vitro*. HDAC10 overexpression promoted tube formation, whereas HDAC10 depletion significantly suppressed tube formation (Figure 1D). Additionally, HDAC10 overexpression increased the number of tubes and crossing points, whereas HDAC10 depletion decreased these values (Figure 1E).

Next, we performed a Matrigel plug assay with cells transfected with an HDAC10 expression vector (FUW-HDAC10), a control vector (FUGW), two different shHDAC10 vectors or a scrambled shRNA control vector to further investigate the function of HDAC10 in angiogenesis *in vivo*. Consistent with the *in vitro* studies, the Matrigel plug assay showed that HDAC10 overexpression promoted angiogenesis and HDAC10 depletion inhibited angiogenesis *in vivo* (Figure 1F). We also generated paraffin sections of the plugs and performed immunostaining with an anti-CD31 antibody. The HDAC10-overexpressing group exhibited a significantly greater number of blood vessels than the control (Figure 1G). In contrast, the HDAC10-depleted group exhibited a profound decrease in the number of blood vessels compared with the scrambled control (Figure 1G). Compared with the control group, the HDAC10-overexpressing group exhibited a more than two-fold increase in blood vessels, and both shHDAC10-depleted groups showed a greater than 50% decrease in the number of blood vessels compared with that in the scrambled control (Figure 1H). Thus, HDAC10 is critically involved in promoting angiogenesis both *in vitro* and *in vivo*.

### HDAC10 regulated target genes in endothelial cells

**Figure 2:**
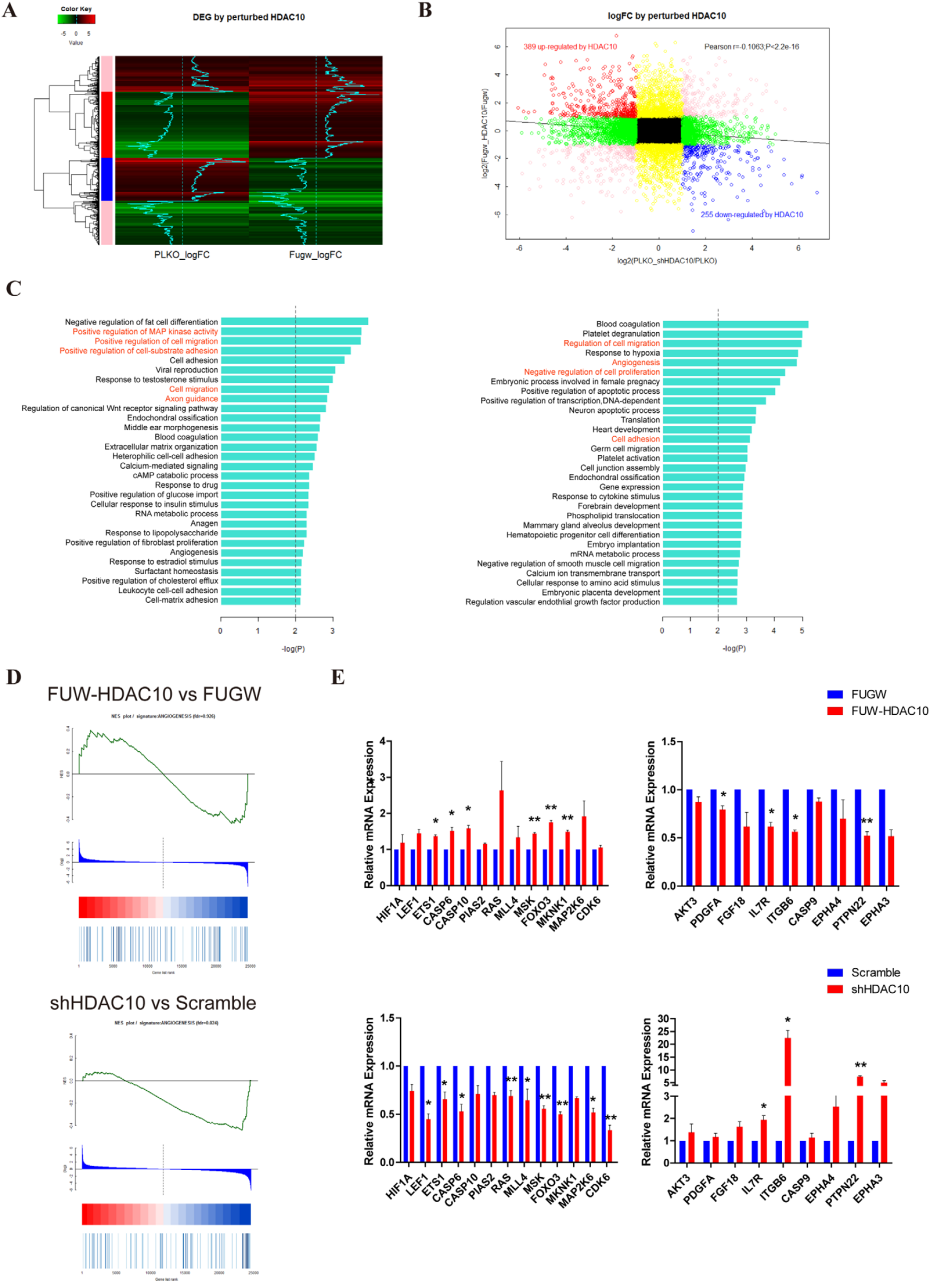
HDAC10 significantly regulates angiogenesis-related genes **(A)** A heat map showing the top 1,000 genes regulated by HDAC10 with the most significant changes in the microarray RNA chip data. **(B)** The group in black represents genes with less than 2-fold changes in gene expression; The group in yellow represents 2-fold or more changes in gene expression in response to HDAC10 overexpression but less than 2-fold changes in gene expression in response to shHDAC10; the group in green represents 2-fold or more changes in gene expression in response to shHDAC10 but less than 2-fold changes in gene expression in response to HDAC10 overexpression; and the group in pink represents that the fold change in gene expression occurs in the same direction in response to HDAC10 overexpression or shHDAC10, although a greater than 2-fold change was observed. The group in red represents a greater than 2-fold change in gene expression among the two datasets, in which the expression was positively regulated by HDAC10 and negatively regulated by shHDAC10. In contrast, the group represents that gene expression was negatively regulated by HDAC10 and positively regulated by shHDAC10. **(C)** Biological functions affected by HDAC10 (left panel) and shHDAC10 (right panel). **(D)** GESA analysis of the angiogenesis-related genes regulated by HDAC10 and shHDAC10. **(E)** Real-time PCR confirmed the differentially regulated genes in the microarray RNA data. Data are presented as the means ± SEM. *P < 0.05 and **P < 0.01 compared with the control or scrambled control.

Next, we performed a microarray analysis using the FUGW, FUW-HDAC10, PLKO and shHDAC10 cells to identify the target genes regulated by HDAC10 in endothelial cells. The heat map shows 1,000 of the differentially expressed genes that exhibit the greatest changes in the microarray data (Figure 2A). A Pearson plot analysis comparing differentially expressed genes with a greater than 2-fold change revealed that 389 genes were up-regulated and that 255 genes were down-regulated by HDAC10 (Figure 2B). According to a Gene Ontology (GO) term analysis, HDAC10 regulated multiple biological functions involved in angiogenesis, such as cell migration, cell adhesion, and MAPK signaling (Figure 2C). Moreover, the Gene Set Enrichment Analysis (GSEA) statistics showed that HDAC10 depletion sharply down-regulated the expression of angiogenesis-related genes, whereas HDAC10 overexpression up-regulated the expression of many angiogenesis-related genes in endothelial cells (Figure 2D). In addition, we performed real-time PCR to confirm our microarray data, and the results suggest that HDAC10 regulates angiogenesis (Figure 2E). These results suggest that HDAC10 is an important regulator of angiogenesis-related gene expression in endothelial cells.

### ERK1/2 is downstream of HDAC10 in the pathway regulating angiogenesis

**Figure 3:**
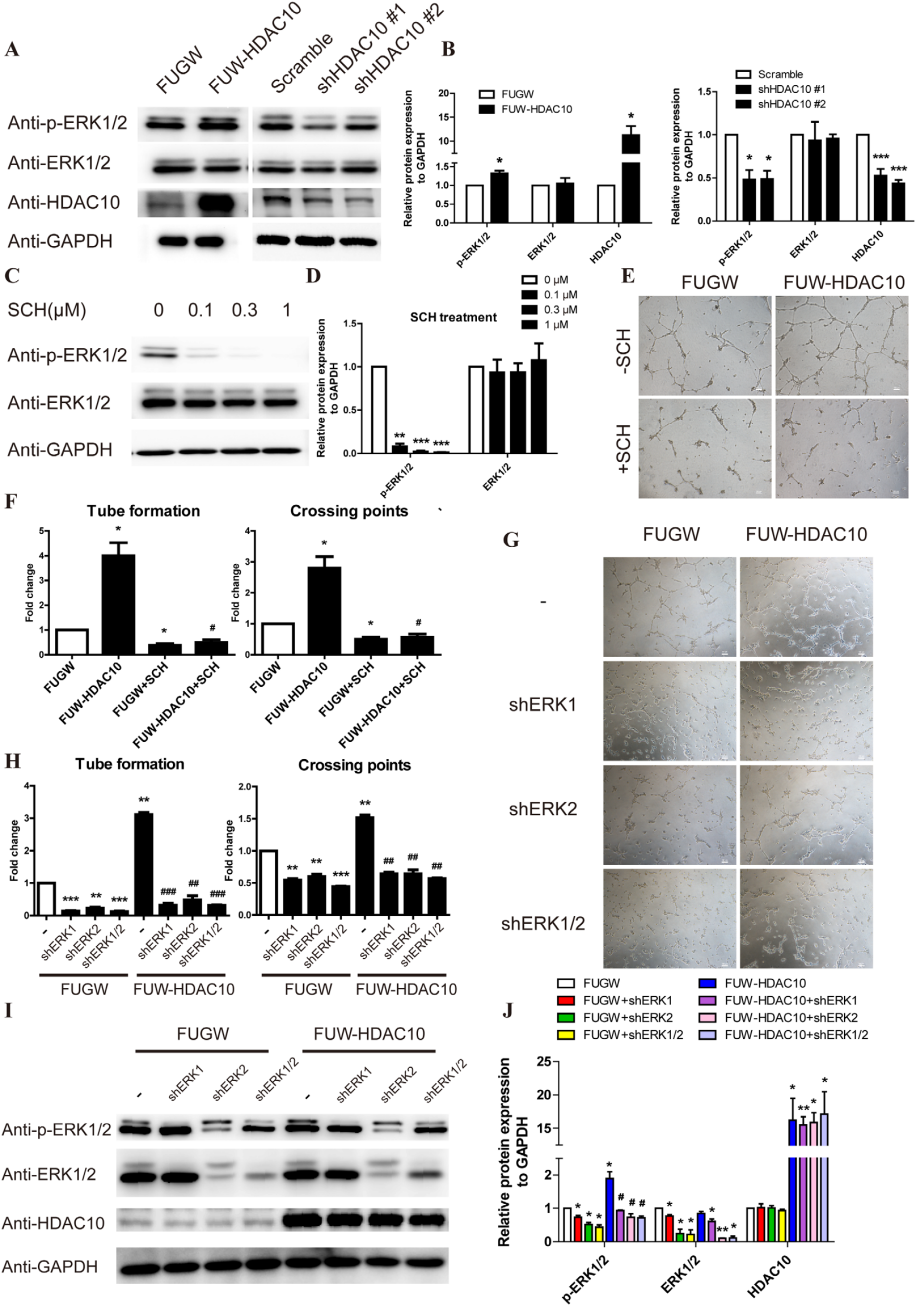
ERK1/2 functions downstream of HDAC10 in the pathway regulating angiogenesis **(A)** Western blot analysis of phosphorylated ERK1/2 levels in HUVECs infected with FUGW, FUW-HDAC10, scrambled control, shHDAC10#1 or shHDAC10#2 lentiviruses. **(B)** Quantitative analysis of the Western blots in **(A)**. **(C)** Western blot analysis of phosphorylated ERK1/2 levels in HUVECs that were pretreated with DMSO, 0.1, 0.3, and 1 μM SCH772984 (SCH). **(D)** Quantitative analysis of the Western blots shown in panel C. **(E)** Inhibitory effect of 0.3 μM SCH on HDAC10-induced HUVEC tube formation *in vitro*. **(F)** Quantitative analysis of the tubes and crossing points shown in panel **E** (n = 5). **(G)** Effects of shERK1, shERK2 and shERK1/2 on HDAC10-induced HUVEC tube formation *in vitro*. **(H)** Quantitative analysis of the tubes and crossing points shown in panel G (n = 5). **(I)** Western blot analysis of phosphorylated ERK1/2 levels in HDAC10-induced tubes formed by HUVECs that were transfected with shERK1, shERK2 and shERK1/2 *in vitro*. **(J)** Quantitative analysis of the Western blots shown in panel **I**. Data are presented as the means ± SEM. *P < 0.05, **P < 0.01 and ***P < 0.001 compared with the control or scrambled control; #*P* < 0.05, ##*P* < 0.01 and ###*P* < 0.001 compared with FUW-HDAC10.

To gain further insight into the mechanism by which HDAC10 regulates angiogenesis, we analyzed the effects of HDAC10 overexpression and knockdown on MAPK activation, which plays crucial roles in the regulation of endothelial cell angiogenesis. HDAC10 overexpression increased the level of activating ERK1/2 phosphorylation, whereas HDAC10 knockdown decreased ERK1/2 phosphorylation (Figure 3A-3B). We then examined whether treating the cells with SCH772984, a specific ERK1/2 inhibitor [36], could inhibit angiogenesis induced by HDAC10 overexpression. As expected, ERK1/2 phosphorylation in endothelial cells was efficiently inhibited with 0.3 μM SCH (Figure 3C-3D). Importantly, the angiogenesis-promoting effects of HDAC10 were completely blocked by the ERK1/2 inhibitor (Figure 3E-3F).

We next generated viral vectors expressing shRNAs for ERK1 and ERK2 and infected endothelial cells with them to further study the role of ERK1/2 in HDAC10-mediated angiogenesis. Both the ERK1 and ERK2 shRNAs blocked the angiogenic function of HDAC10 in endothelial cells (Figure 3G-3H). Western blot analysis showed that the activating ERK1/2 phosphorylation was also inhibited (Figure 3I-3J). Collectively, these results strongly suggest that HDAC10 controls endothelial cell angiogenesis through regulation of ERK1/2 signaling.

### HDAC10 binds to the promoter regions of PTPN22 and deacetylates histones H3 and H4 in these regions

**Figure 4:**
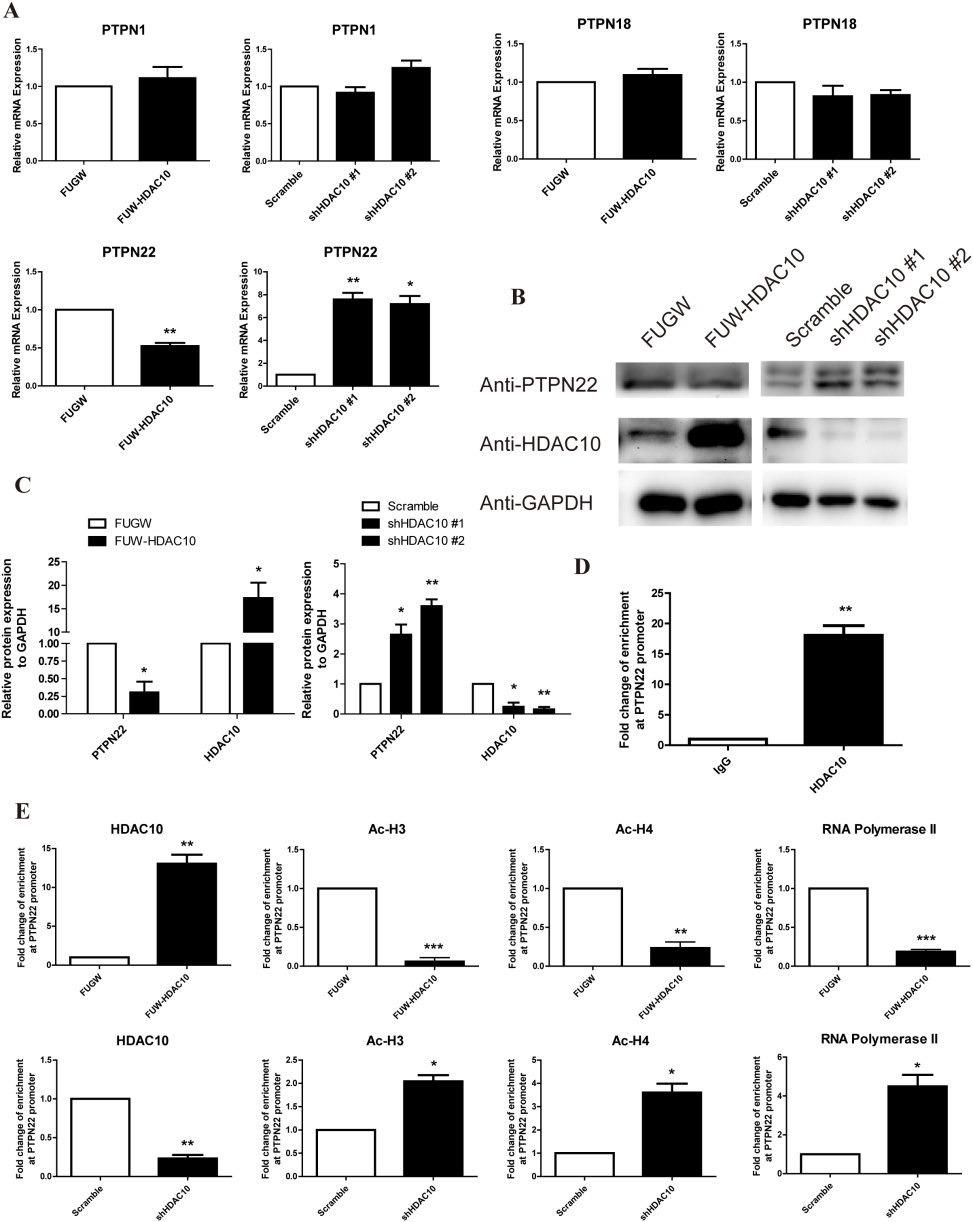
HDAC10 up-regulates ERK1/2 phosphorylation by inhibiting PTPN22 **(A)** Real-time PCR analysis shows that HDAC10 regulates the mRNA levels of several members of the PTPN family. **(B)** Western blot analysis showing that HDAC10 regulates PTPN22 protein expression. **(C)** Quantitative analysis of the Western blots shown in panel **B**. **(D, E)** Results of the chromatin immunoprecipitation assay (ChIP assay). **(D)** HDAC10 binds to the PTPN22 promoter. **(E)** HDAC10 decreases the acetylation of histones H3 and H4 at the PTPN22 promoter. Acetylated histone H3 and H4 antibodies were used to precipitate DNA in the ChIP assay. Primers that target the transcription factor binding sites in the PTPN22 promoter were used for quantitative real-time PCR. The data were obtained from three independent experiments. Reduced acetylation of H3 and H4 blocks the binding of RNA polymerase II to the transcription factor binding sites. DNA was precipitated from HUVECs in the control group and the HDAC10 overexpression group using an anti-RNA polymerase II antibody in the ChIP assay. Data are presented as the means ± SEM. *P < 0.05, **P < 0.01 and ***P < 0.001 compared with the control or scrambled control.

Based on the microarray data and other results described above, we hypothesize that HDAC10 regulates the expression of PTPN22, which in turn regulates ERK1/2 phosphorylation [17, 18, 37]. Thus, we analyzed the expression levels of PTPN22 and several other members of the PTPN family, such as PTPN1 and 18, in HDAC10 overexpression and knockdown cells using real-time PCR. The level of PTPN22 mRNA, but not those of the other PTPNs, was dramatically regulated by HDAC10 (Figure 4A). Western blot analysis confirmed that HDAC10 overexpression down-regulated PTPN22 expression, whereas HDAC10 knockdown up-regulated PTPN22 expression (Figure 4B-4C). Because acetylation is a key factor for regulating gene expression, we hypothesized that HDAC10 binds the PTPN22 gene promoter, thus regulating its acetylation and gene expression. To test this hypothesis, we first employed the chromatin immunoprecipitation (ChIP) assay to investigate whether HDAC10 binds to the PTPN22 gene promoter. The primers used in the ChIP assay were designed to target the binding sites for transcription factors in the PTPN22 gene promoter. The results of the ChIP assay showed that HDAC10 indeed bound to the promoter region of the PTPN22 gene (Figure 4D). We next investigated the effects of HDAC10 on the acetylation of histones H3 and H4 and on the binding of RNA polymerase II to the PTPN22 gene promoter using the ChIP assay. The results showed that acetylation of histones H3 and H4 and the binding of RNA polymerase II to the PTPN22 gene promoter were significantly reduced in cells overexpressing HDAC10 (Figure 4E). In contrast, HDAC10 knockdown increased the acetylation of histone H3 and H4 and the binding of RNA polymerase II to the PTPN22 gene promoter (Figure 4E). These results provide strong evidence supporting our hypothesis that HDAC10 binds the PTPN22 gene promoter and consequently regulates its acetylation and gene expression.

### PTPN22 is required for HDAC10-mediated regulation of ERK1/2 phosphorylation and angiogenesis

**Figure 5:**
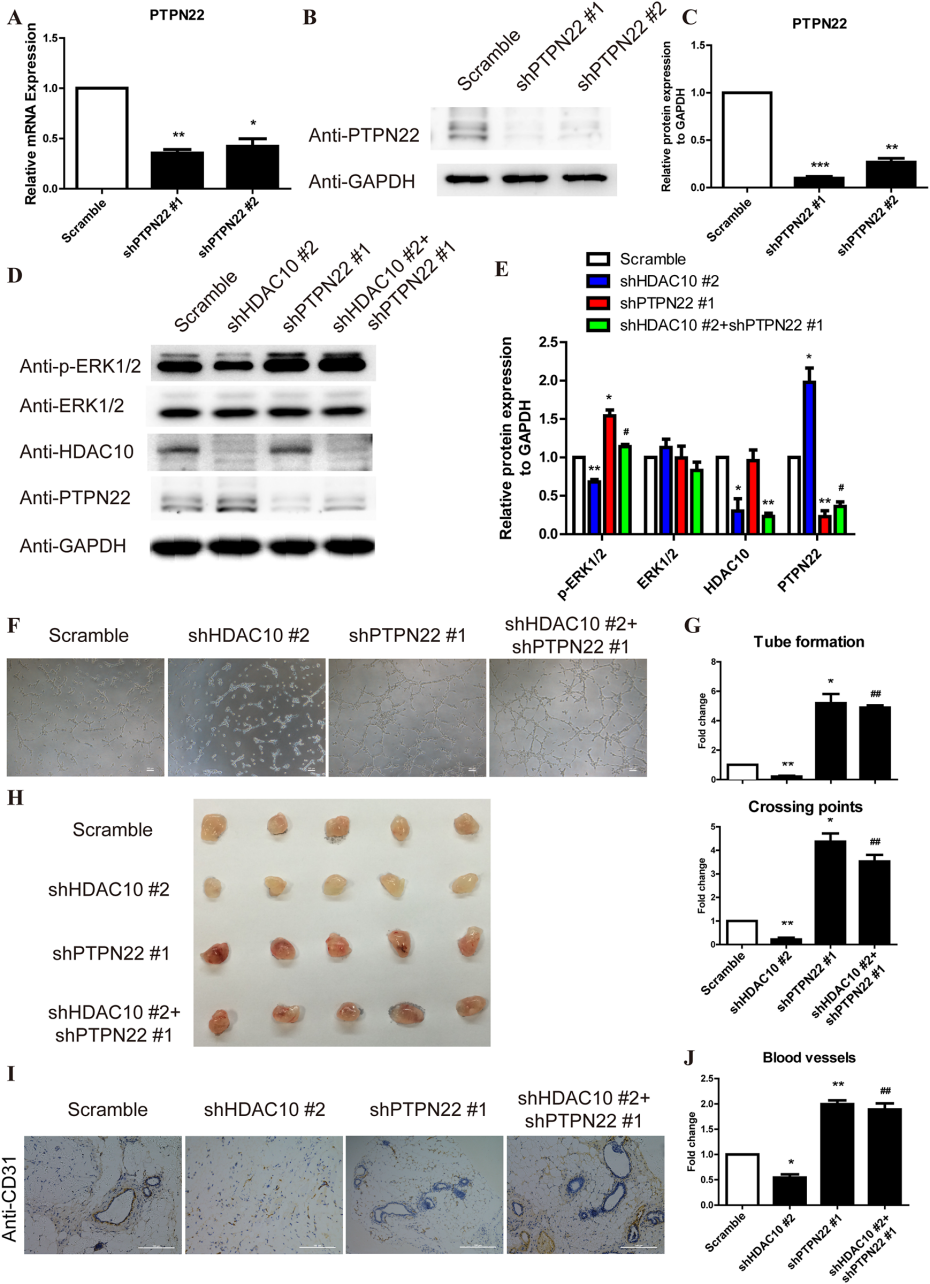
The PTPN22/ERK axis is the critical target of HDAC10 to promote angiogenesis **(A)** HUVECs were infected with scrambled control, shPTPN#1 and shPTPN#2 lentiviruses. PTPN22 mRNA expression was analyzed by real-time PCR. **(B)**. PTPN22 protein expression was analyzed by Western blotting. **(C)** Quantitative analysis of the Western blots shown in panel **B**. **(D)** HUVECs were infected with shHDAC10 and co-infected with scrambled control, shPTPN#1 or shPTPN#2. Western blot analysis of the phosphorylated ERK1/2 levels in each group. **(E)** Quantitative analysis of the Western blots shown in panel **D**. **(F)** Effect of shPTPN22 up-regulation on shHDAC10-induced inhibition of HUVEC tube formation *in vitro*. **(G)** Quantitative analysis of the tubes and crossing points shown in panel **F** (n = 5). **(H)** The effects of shPTPN22 on the shHDAC10-induced inhibition of *in vivo* angiogenesis were assessed using a Matrigel plug assay. Photographs of representative Matrigel plugs show vessel growth and CD34 expression **(I)**. n = 5 per group. **(J)** The blood vessels were quantified by manually counting the images shown in **(I)**, and the data are presented as a percentage of the control (n = 5). Data are presented as the means ± SEM. *P < 0.05, **P < 0.01 and ***P < 0.001 compared with the scrambled control; #*P* < 0.05, ##*P* < 0.01 compared with shHDAC10.

To further investigate the function of PTPN22 in the HDAC10 regulation of ERK1/2 phosphorylation and angiogenesis, we generated vectors expressing two different shRNAs for PTPN22 and established PTPN22 knockdown (shPTPN22) (Figure 5A-5C) and PTPN22 and HDAC10 double knockdown (shPTPN22/shHDAC10) (Figure 5C-5D) cells. As shown in Figure 5C-5D, shPTPN22 not only promoted ERK1/2 phosphorylation but also rescued the inhibition of ERK1/2 phosphorylation induced by shHDAC10. Similarly, shPTPN22 also enhanced angiogenesis and relieved the shHDAC10-induced inhibition of angiogenesis (Figure 5E). The statistical analyses of the tube numbers and crossing points are shown in Figure 5F.

We also used the Matrigel assay to investigate the effect of shPTPN22 on the ability of endothelial cells to form blood vessels *in vivo*. The shHDAC10-induced inhibition of angiogenesis was markedly reversed by shPTPN22 (Figure 5G). As shown in the immunohistochemical staining, the number of blood vessels of the shPTPN22/shHDAC10 group was increased compared with the shHDAC10 group (Figure 5H-5I). Collectively, these results suggest that PTPN22 is critical for HDAC10-mediated regulation of ERK1/2 phosphorylation and angiogenesis.

## DISCUSSION

Angiogenesis is an important event in many physiological and pathological processes including tumor growth. Recent studies have suggested that HDACs may play important roles in regulation of angiogenesis [5, 24–26], suggesting that HDACs could potentially serve as important therapeutic targets for modulation of angiogenesis. However, it appears that different members of the HDAC family can exert different and even opposing roles in this process [5, 24–26, 28–29, 38]. Thus, it is critically important to define the role of specific HDACs in angiogenesis and determine the underlying mechanisms. In the current study, we have identified HDAC10, a member of the HDAC II family [36], as a key regulator of angiogenesis. Using both overexpression and knockdown strategies, we demonstrate that the level of HDAC10 in endothelial cells is crucial for regulation of angiogenesis: a higher level of HDAC10 promotes whereas a lower level of HDAC10 inhibits angiogenesis.

How does HDAC10 regulate angiogenesis? Our results show that HDAC10 regulates angiogenesis by controlling ERK1/2, a key signaling intermediate in the cellular control of angiogenesis [9, 10, 39] Specifically, HDAC10 overexpression in endothelial cells increased the level of phosphorylated ERK1/2, whereas depletion of HDAC10 in endothelial cells reduced the level of phosphorylated ERK1/2. Moreover, using an ERK1/2 inhibitor, we showed that ERK1/2 activity is indispensable for HDAC10-induced angiogenesis. These findings reveal a novel regulator of ERK1/2 activity and, hence, angiogenesis in endothelial cells.

How does HDAC10 regulate ERK1/2 activity? A main function of HDACs is to remove acetyl groups from the N-acetyl lysines on histones, thereby modifying the chromatin structure and modulating gene transcription [22]. Thus, a potential mechanism by which HDAC10 regulates ERK1/2 activity is by modulating the expression of genes that are critical for ERK1/2 activation. To test this hypothesis, we analyzed the effects of HDAC10 on gene expression using an RNA expression array. These studies revealed that HDAC10 negatively regulated the expression of PTPN22, which is a member of PTPN family of non-receptor type PTPNs [40–42]. Furthermore, we showed that HDAC10 bound to the PTPN22 promoter and promoted the deacetylation of histones H3 and H4, which reduced the binding of RNA polymerase II to the promoter of the PTPN22 gene and consequently inhibited PTPN22 transcription. Additionally, using shRNAs targeting PTPN22, we showed that PTPN22 was indeed critical for mediating HDAC10-induced regulation of ERK activation and angiogenesis.

In summary, the studies presented in this paper have revealed a novel signaling pathway consisting of HDAC10, PTPN22 and ERK1/2 that is critical for regulating angiogenesis. Our results suggest that HDAC10 regulates angiogenesis by controlling the acetylation of the promoter region of PTPN22 and thus the binding of RNA polymerase II to this region and PTPN22 gene expression. The change of PTPN22 expression in turn regulates ERK1/2 phosphorylation and consequently the angiogenic activity of endothelial cells. Given the importance of angiogenesis in a variety of physiological and pathological processes including tumor growth, the HDAC10-PTPN22-ERK1/2 signaling axis may provide a useful target for the development of novel therapeutic strategies to control diseases associated with abnormal angiogenesis.

## MATERIALS AND METHODS

### Cell culture

HUVECs were purchased from the Cell Bank at the Shanghai Institutes for Biological Sciences of the Chinese Academy of Sciences and cultured in RPMI-1640 medium (GIBCO) supplemented with 10% fetal calf serum (GIBCO), 100 U/ml streptomycin and 100 U/ml penicillin (GIBCO). Human embryonic kidney 293T cells (HEK293FT) were purchased from the American Type Culture Collection (ATCC) and cultured in Dulbecco’s modified Eagle’s medium (DMEM, GIBCO) supplemented with 10% fetal calf serum, 100 U/ml streptomycin and 100 U/ml penicillin.

### Plasmid construction

The GFP-expressing FUGW lentivirus vector was previously described [35]. The coding sequence of wild-type human HDAC10 was amplified from HUVEC cDNA by PCR. The FUGW lentivirus vector was cleaved with Age I/EcoRI restriction enzymes and the GFP coding sequence was replaced with that of HDAC10 (termed as FUW-HDAC10). The shRNA constructs targeting scramble, HDAC10, PTPN22 and ERK1/2 were synthetized by JieRui BioTech Company and are as follows: scramble, CCTAAGGTTAAGTCGCCCTCG; HDAC10, TCACTGCACTTGGGAAGCTCCTGTA and CGGGTTCTGTGTGTTCAACAA; PTPN22, TATGATCACAGTCGTGTTAAA and AGAAGATGTGAAGTCGTATTA; ERK1, CGTGCTCCACCGAGATCTAAA and GACAGACATCTCTGCACCCTG; and ERK2, CCCATATCTGGAGCAGTATTA and TATCCATTCAGCTAACGTTCT. The annealing shRNA constructs were cloned into pLKO.1 lentiviral vector via the AgeI and EcoRI restriction sites.

### Virus generation and infection

To generate the lentivirus, FUGW, FUW-HDAC10 and shRNA constructs were co-transfected with helper plasmids into HEK293T cells with the X-tremeGENE HP DNA transfection reagent (Roche). At 48 h post-transfection, the virus was harvested. HUVECs were plated at a density of 1.2 × 10^5^ cells/well of 6-well plate, grown to 30% to 40% confluence and then infected with the virus.

### Reverse transcription and real-time PCR

Total RNAwas isolated from HUVECs using TRIzol (Invitrogen), and 500 ng of RNA from each sample was reverse-transcribed into cDNA using the PrimeScript™ RT reagent kit (TaKaRa). The cDNA was used as the template for real-time PCR.

### Antibodies and Western blot

The antibodies used in this study are as follows: HDAC10 (Sigma, H3413), ERK1/2 (Cell Signaling Technology, #4695), p-ERK1/2 (Thr202/Tyr204) (Cell Signaling Technology, #4370), PTPN22 (Cell Signaling Technology, #14693), and GAPDH (Santa Cruz Biotechnology, sc-47724). For Western blot, HUVECs were lysed with radio-immunoprecipitation assay buffer (50 mM Tris, 0.1% sodium dodecyl sulfate, 1.0% IGEPAL CA-630, 150 mM NaCl, and 0.5% sodium deoxycholate, pH 8.0, The Shanghai Sheng Gong Company) on ice for 25 min. The lysates were centrifuged at 12,000 rpm for 15 minutes at 4°C, and the protein concentrations in the samples were determined using the bicinchoninic (BCA) method. Equal amounts of protein were loaded onto SDS-PAGE gels, separated by electrophoresis, transferred to membranes and incubated with the appropriate antibodies. Then, the signals were developed using the Clarity Western enhanced chemiluminescence (ECL) substrate (1705061, Bio-Rad).

### Tube formation assay

HUVECs (as specified in each experiment) were plated at a density of 4 × 10^4^ cells/well in 24-well plates (Corning, Germany) that were pre-coated with 330 μl of Matrigel Growth Factor Reduced Membrane Matrix (BD Biosciences). Tube numbers and crossing points were quantified in 5 random microscopic fields after 8 h.

### Matrigel plug assay

HUVECs (as specified in each experiment) were suspended in Matrigel and subcutaneously injected into the backs of 8-week-old nude mice (each mouse was injected with one Matrigel plug) [43]. For each experiment, five mice were injected with each type of HUVECs (i.e., HDAC10-overexpressing, HDAC10 knockdown or control). All experiments were repeated three times. After 7 days, the Matrigel plugs were removed to visualize the capillaries. Plug sections were stained with a platelet endothelial cell adhesion molecule-1 (PECAM-1) antibody (M-20) (sc-1506, Santa Cruz Biotechnology), and nuclei were stained with Hoechst 33342. The level of angiogenesis *in vivo* was quantified by counting the number of vessels in 5 random microscopic fields.

### ChIP assay

HUVECs were harvested with 0.25% trypsin (GIBCO) and cross-linked for 10 minutes by directly adding 1% formaldehyde, which was then neutralized with 0.125 M glycine for 5 minutes with gentle shaking. The cells were washed with cold PBS and lysed for 15 minutes on ice in a cell lysis solution (5 mM PIPES, pH 8.0, 85 mM KCl_2_, and 0.5% NP-40) to obtain cell nuclei. The nuclei were lysed in nuclei lysis buffer (50 mM Tris-Cl, pH 8.1, 10 mM EDTA, and 1% SDS) for an additional 30 minutes and gently pelleted for 10 minutes on ice. The chromatin was sonicated and sheared using a sonicator (Focused-ultrasonicator, M220, COVARIS). The sheared chromatin samples were used as an input control or incubated with antibodies overnight; 20 μl of beads was added, and the samples were incubated for 1.5 h rotating at 15 rpm. Then, the samples were successively washed 4 times with TSE I (0.1% SDS, 2 mM EDTA, 20 mM Tris-Cl, pH 8.1, 150 mM NaCl, and 1% Triton X-100), TSE II (0.1% SDS, 2 mM EDTA, 20 mM Tris-Cl, pH 8.1, 300 mM NaCl, and 1% Triton X-100), wash buffer (0.25 M LiCl, 1 mM EDTA, 10 mM Tris-Cl, pH 8.1, 1% NaDOC, and 1% NP400), and TE buffer (1 mM EDTA and 10 mM Tris-Cl). The DNA samples were dissociated from the immunoprecipitated chromatin and used as templates for real-time PCR with primer pairs targeting the PTPN22 promoter.

### Immunohistochemistry

Ten-micrometer-thick, formalin-fixed paraffin-embedded Matrigel plug sections were stained with a goat monoclonal antibody against CD31 (Santa Cruz Biotechnology, sc-71872, 1:100 dilution). Images were captured from randomly selected fields.

### Statistical analysis

All data represent as mean ± SEM. 2-tailed Student’s t test (for parametric data) or Mann-Whitney test (for nonparametric data) were used to compare two groups of samples according to distribution. P values less than 0.05 were considered significant. Prism 5 (GraphPad) was used for statistical analysis.
